# The complete chloroplast genome sequence of *Camellia rostrata* S. X. Yang & S. F. Chai (Theaceae), a critically endangered yellow camellia from southwest China

**DOI:** 10.1080/23802359.2021.1955028

**Published:** 2021-07-19

**Authors:** Zhao-Yuan Zhang, Ye-Kun Yang, Pin-Ming Ye, Jin-Lin Ma, Hang Ye

**Affiliations:** a Guangxi Key Laboratory of Special Non-wood Forest Cultivation and Utilization, Guangxi Forestry Research Institute, Nanning, Guangxi, China; b Golden Camellia Park, Nanning, Guangxi, China

**Keywords:** *Camellia rostrata*, chloroplast genome, illumina sequencing, phylogenetic analysis

## Abstract

*Camellia rostrata* S. X. Yang & S. F. Chai is a recently described yellow camellia species from Guangxi, China. It is a critically endangered species according to the IUCN Red List Categories and Criteria. Here, we report the complete chloroplast (cp) genome based on next-generation sequencing technology. The complete cp genome of *C. rostrata* is 156,547 bp in length and consists of a large single-copy (LSC, 86,199 bp) region, a small single-copy (SSC, 18,204 bp) region, and a pair of inverted repeats (IRs, 26,072 bp). The genome contains 135 genes including 40 tRNA, eight rRNA, and 87 protein-coding genes. Phylogenetic analysis resolved *C. rostrata* in a clade containing *C. huana* and *C. impressinervis*, both of which are classified to *Camellia* sect. *Archecamellia.* Our findings support the placement of *C. rostrata* in *C.* sect. *Archecamellia* as proposed by a previous study. The cp genome of *C. rostrata* provides valuable bioinformatic resources for the protection and utilization of this yellow camellia species.

*Camellia* contains ca. 120 species (Ming 2000; Ming and Bartholomew 2007). It is mainly distributed in East and Southeast Asia, with a diversity centered in the Southern Yangtze River of China (Ming and Zhang 1996). One of these species, *C. rostrata* S. X. Yang & S. F. Chai is a newly described yellow species of *Camellia* from Guangxi, China (Liu et al. 2020). This species is restricted in its distribution to its type locality, which reportedly has <100 individuals, and was thus proposed as a critically endangered species according to the IUCN Red List Categories and Criteria (Liu et al. 2020). In this study, we report the complete cp genome sequence of *C. rostrata*, and constructed the phylogenetic relationship between *C. rostrata* and other congeneric species. The cp genome of *C. rostrata* provides useful bioinformatics for the conservation of this wild yellow species of *Camellia*.

Young leaves of *C. rostrata* were collected from Long’an county of Guangxi in China (23°03′58″N, 107°43′49″E). The voucher specimen (S. X. Yang & F. Y. Wu 6081) was deposited in the Herbarium at Kunming Institute of Botany (KUN), Chinese Academy of Sciences (KUN 1482804, http://www.kun.ac.cn, Jing-Hua Wang, wangjh@mail.kib.ac.cn). Total genomic DNA was extracted using a modified hexadecyltrimethylammonium bromide (CTAB) approach (Doyle and Doyle 1987). Genome sequencing was performed using Illumina Hi-Seq 2500 platform. The chloroplast genome sequences were assembled using GetOrganelle (Jin et al. 2020) and annotated using PGA (Qu et al. 2019). Phylogenetic analysis of *C. rostrata* and 25 other species classified to *Camellia* as well as two outgroups was performed using RAxML version 8.2.12 (Stamatakis 2014) following a previous study (Yu et al. 2017).

The complete chloroplast genome of *C. rostrata* (GenBank accession number MW755303) was obtained with a total length of 156,547 bp and a mean sequencing depth of 155.2. The GC content of the genome is 37.3%. It consisted of a large single-copy (LSC, 86,199 bp) region, a small single-copy (SSC, 18,204 bp) region, and a pair of inverted repeats (IR, 26,072 bp). A total of 135 genes were annotated, including 87 protein-coding, 40 tRNA, and eight rRNA genes. Similar to cp genomes of other *Camellia* species from GenBank, it showed typical quadripartite structure reported in angiosperms (Shinozaki et al. 1986).

Based on morphological evidence, Liu et al (2020) stated that *C. rostrata* was most closely related to *C. debaoensis* R. C. Hu & Y. Q. Liufu. However, these phylogenetic analyses indicate that *C. rostrata, C. huana*, and *C. impressinervis* grouped in a strongly supported clade (BS = 94%), which is sister to another highly supported clade containing *C. debaoensis* and *C. mingii* S. X. Yang (BS = 87%) (Figure 1). *Camellia huana* and *C. impressinervis* are classified in *C.* sect. *Archecamellia* (Ming and Bartholomew 2007). Thus, our findings supported the placement of *C. rostrata* in *C.* sect. *Archecamellia* as proposed by Liu et al. (2020). Both *C. debaoensis* and *C. mingii* were consistently placed in *C.* sect. *Stereocarpus* (Hu et al., 2019; Liu et al., 2019). *Camellia* sect. *Archecamellia* was not revealed as monophyletic since another one/two species (*C. petelotii*, *C. nitidissima*, the latter was treated as a synonym of the former according to Ming’s classification system) from this section were not nested within the *C. rostrata* clade. The cp genome reported here provides a reference for future study on the phylogenomics of *Camellia*, as well as the protection and utilization of *C. rostrata*.

**Figure 1.**
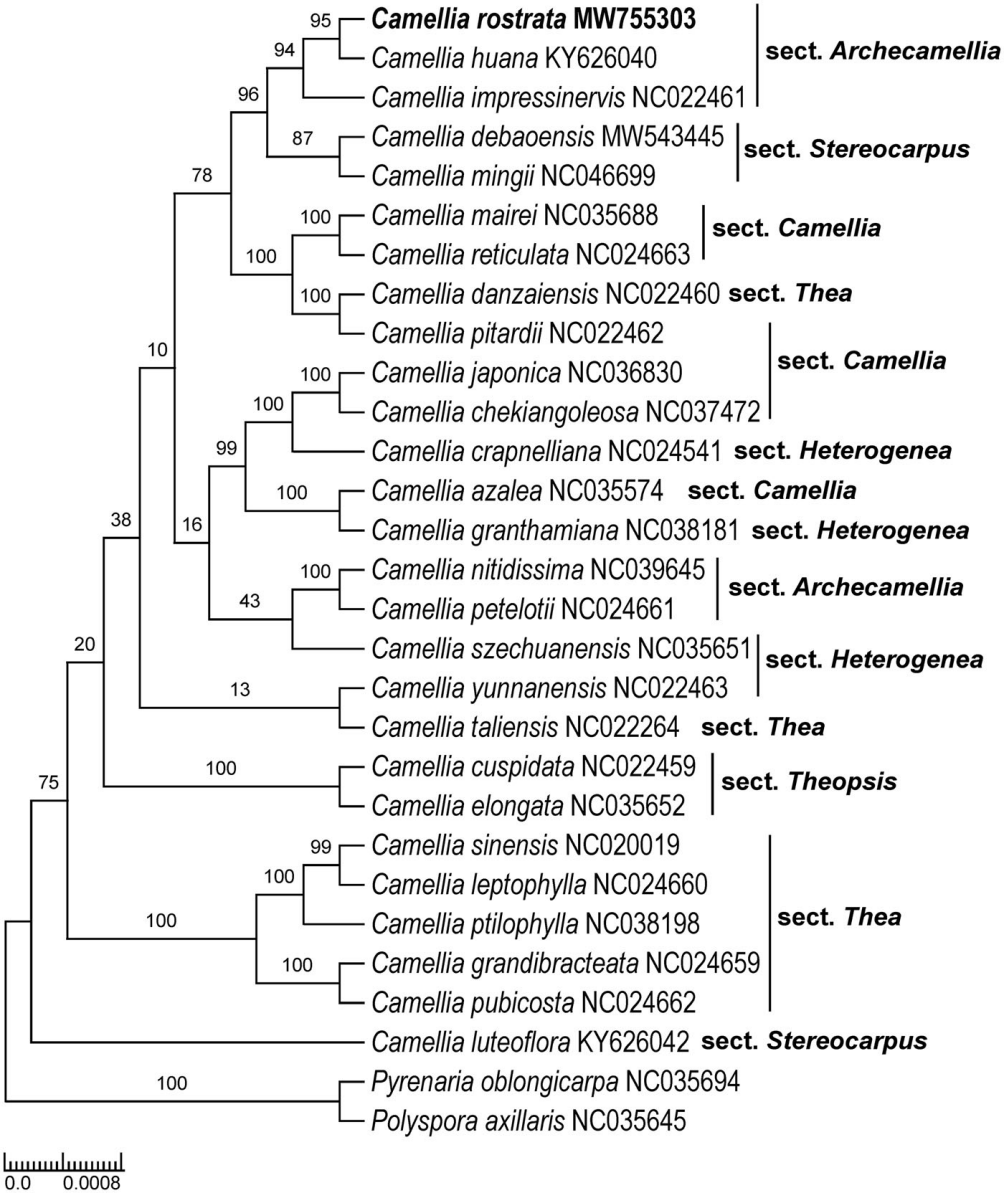
Maximum likelihood tree of Theaceae based on 28 complete chloroplast genome sequences, including *Camellia rostrata* (GenBank accession number is MW755303) sequenced in this study. The bootstrap support values are shown beside the nodes. Two representative taxa of Theaceae (*Polyspora axillaris*, NC035645; *Pyrenaria oblongicarpa*, NC035694) were used as outgroups.

## Data Availability

The data that support the findings of this study are openly available in GenBank of NCBI at https://www.ncbi.nlm.nih.gov/, under the accession no. MW755303. The associated BioProject, SRA, and Bio-Sample numbers are PRJNA725164, SRR14326646, and SRS8774895, respectively.
